# Monitoring and treatment of alcohol use disorder: an integrated multidisciplinary model

**DOI:** 10.20517/mtod.2024.103

**Published:** 2025-03-30

**Authors:** Ashwani K. Singal

**Affiliations:** Department of Medicine, Division of Gastroenterology Hepatology and Nutrition, University of Louisville, Louisville, KY 40202, USA.

**Keywords:** AH, alcohol, ALD, AUD, cirrhosis, depression, LT

## Abstract

Alcohol-associated liver disease (ALD) is one of the most prevalent liver diseases and a leading indication for liver transplantation (LT). Alcohol use disorder (AUD), commonly present in patients with ALD, may also be associated with psychiatric comorbidities such as depression and anxiety. Early identification of ALD and timely treatment of AUD can help prevent the progression to advanced stages of ALD, including cirrhosis and alcoholic hepatitis. However, screening for alcohol use and managing AUD in ALD patients are often not performed due to various barriers at the patient, clinician, and administrative levels. This review highlights an integrated multidisciplinary care model, emphasizing the roles of hepatologists, psychiatrists, addiction counselors, and social workers in providing comprehensive management of both liver disease and AUD. It outlines laboratory assessments, pharmacological and behavioral therapies, and recommended follow-up evaluations by specialists. The goal of this article is to promote team-based comprehensive care for patients with ALD.

## INTRODUCTION

Alcohol-associated liver disease (ALD) is a common etiology of advanced liver disease worldwide, with a significant prevalence in the United States^[1–3]^. Controlling alcohol use is crucial for determining long-term patient outcomes^[4]^. However, patients frequently experience alcohol relapse, which can lead to severe complications such as decompensated cirrhosis, alcohol-associated hepatitis (AH), and post-liver transplantation (LT) complications^[5,6]^. Since self-reported alcohol use may be inaccurate, gathering information from the patient’s friends, relatives, and colleagues is essential. However, the sensitive nature of this information and the relationships these individuals have with the patient must be carefully considered. Emerging biomarkers of alcohol use are becoming viable tools for both initial assessment and ongoing monitoring of alcohol consumption.

Based on the severity of alcohol use disorder (AUD), treatment options include brief intervention, behavioral interventions, and medications for AUD (MAUD). However, in real-world settings, only 10%−30% of ALD patients receive treatment for AUD, with MAUD being used even less often^[7–9]^. Various patient- and clinician-related barriers hinder AUD treatment in ALD patients^[10,11]^. Beyond patient awareness and attitude toward their disease, the stigma associated with AUD and ALD remains a major barrier^[12]^. Healthcare providers must recognize that these patients suffer from two coexisting conditions: ALD and AUD^[3,13]^. A multidisciplinary integrated mode^[14]^, in which specialties collaborate within a unified clinic, may help address several of the barriers and ultimately improve long-term patient outcomes.

In this review, we discuss (1) the diagnosis and monitoring of alcohol use; (2) treatment options for AUD in ALD patients and their benefits; and (3) the role of a multidisciplinary integrated approach in both pre- and post-transplant settings. Additionally, we highlight future research priorities and potential solutions to enhance the monitoring and treatment of AUD in ALD patients.

## SCREENING AND MONITORING OF ALCOHOL USE

**Figure 1.**
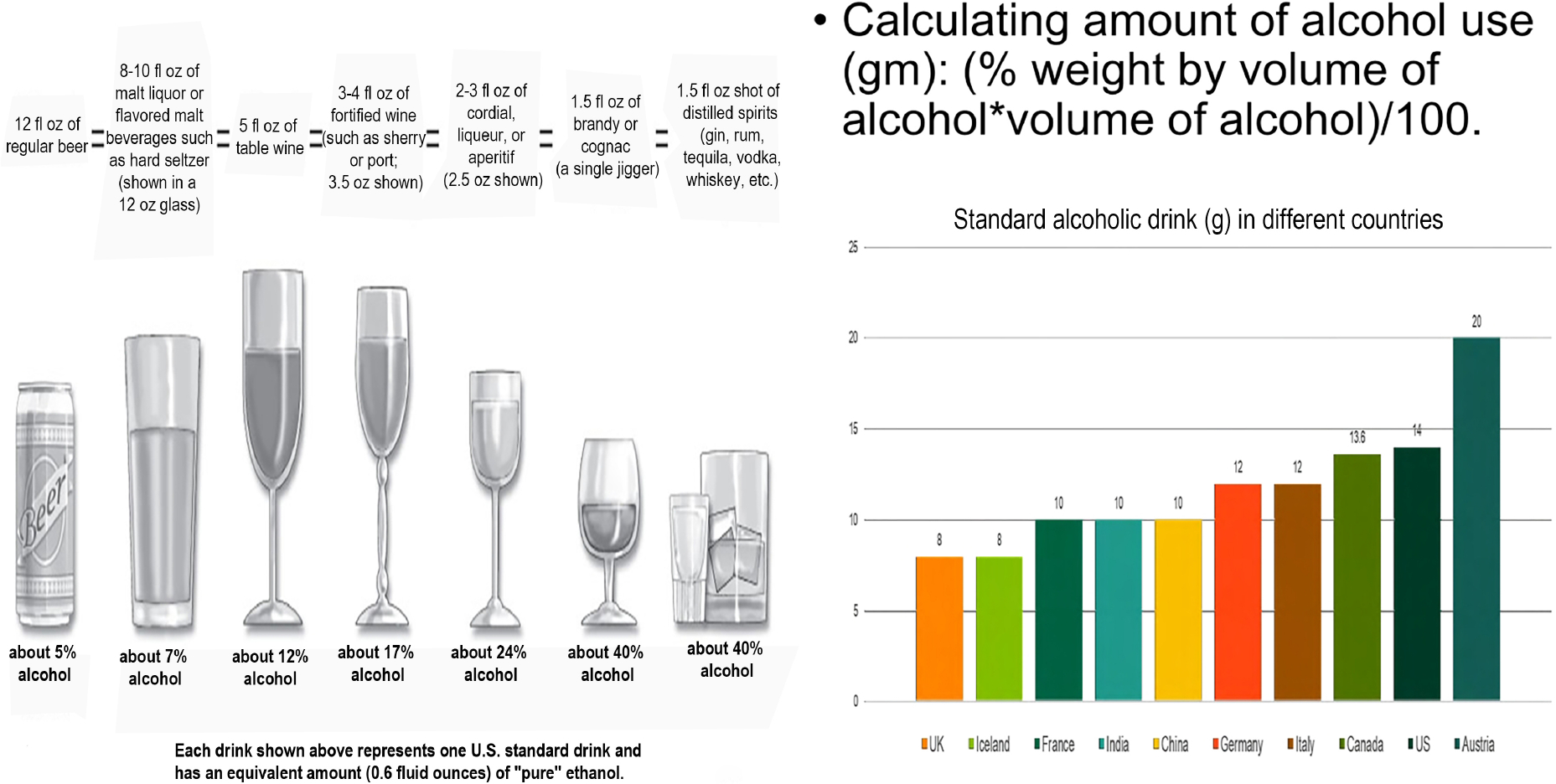
Defining an alcohol-containing drink and quantifying alcohol use.

In the US, heavy alcohol use is defined as consuming ≥ 7 drinks/week or > 3 drinks per occasion for females and ≥ 14 drinks/week or > 4 drinks per occasion for males^[1]^. It is crucial to gather detailed information on the type and volume of alcohol consumed to accurately calculate the total intake of pure alcohol in grams [Figure 1]. Heavy alcohol consumption can result in multiple systemic comorbidities and diseases, with ALD being the most common form of end-organ damage and liver cirrhosis occurring in 10%−20% of affected individuals^[1,15]^.

AUD, as defined by the Diagnostic and Statistical Manual of Mental Disorders, Fifth Edition (DSM-5), is not based on the amount of alcohol consumed but rather on patterns of alcohol use that lead to physical, social, or interpersonal impairments. AUD is diagnosed when an individual meets at least 2 of the 11 criteria outlined in the DSM-5 [Table 1], with severity increasing as more criteria are met^[16]^. The healthcare burden of AUD, including its impact on mental and physical health - particularly its association with ALD - has reached alarming levels. This increase is particularly pronounced among younger people, females, and ethnic minorities^[2,17–19]^. Additionally, the combined use of alcohol and other recreational substances, along with poor dietary habits (e.g., junk foods), exacerbates liver damage in young people^[20]^.

### Significance of screening for AUD

Compared to liver diseases caused by non-alcohol-related factors, ALD patients often present at an advanced stage and experience more rapid disease progression^[21]^. Therefore, healthcare providers should screen for alcohol use at every possible medical encounter. Individuals engaging in harmful alcohol use should be assessed for ALD^[22]^. Interventions to reduce alcohol intake in these individuals can help lower the risk of advanced ALD, including cirrhosis and AH^[23]^. However, due to time constraints, hepatologists and gastroenterologists must prioritize ALD-related health issues in their patients, limiting their ability to address AUD comprehensively - particularly among those with advanced ALD, such as decompensated cirrhosis and AH^[24]^. Moreover, some healthcare providers may feel unprepared to manage AUD due to insufficient formal training in addiction medicine^[10,24]^. These challenges, along with other patient- and system-related barriers, result in very low rates of AUD treatment among patients with ALD.

### Screening tools

#### Self-reported alcohol use

**Figure 2.**
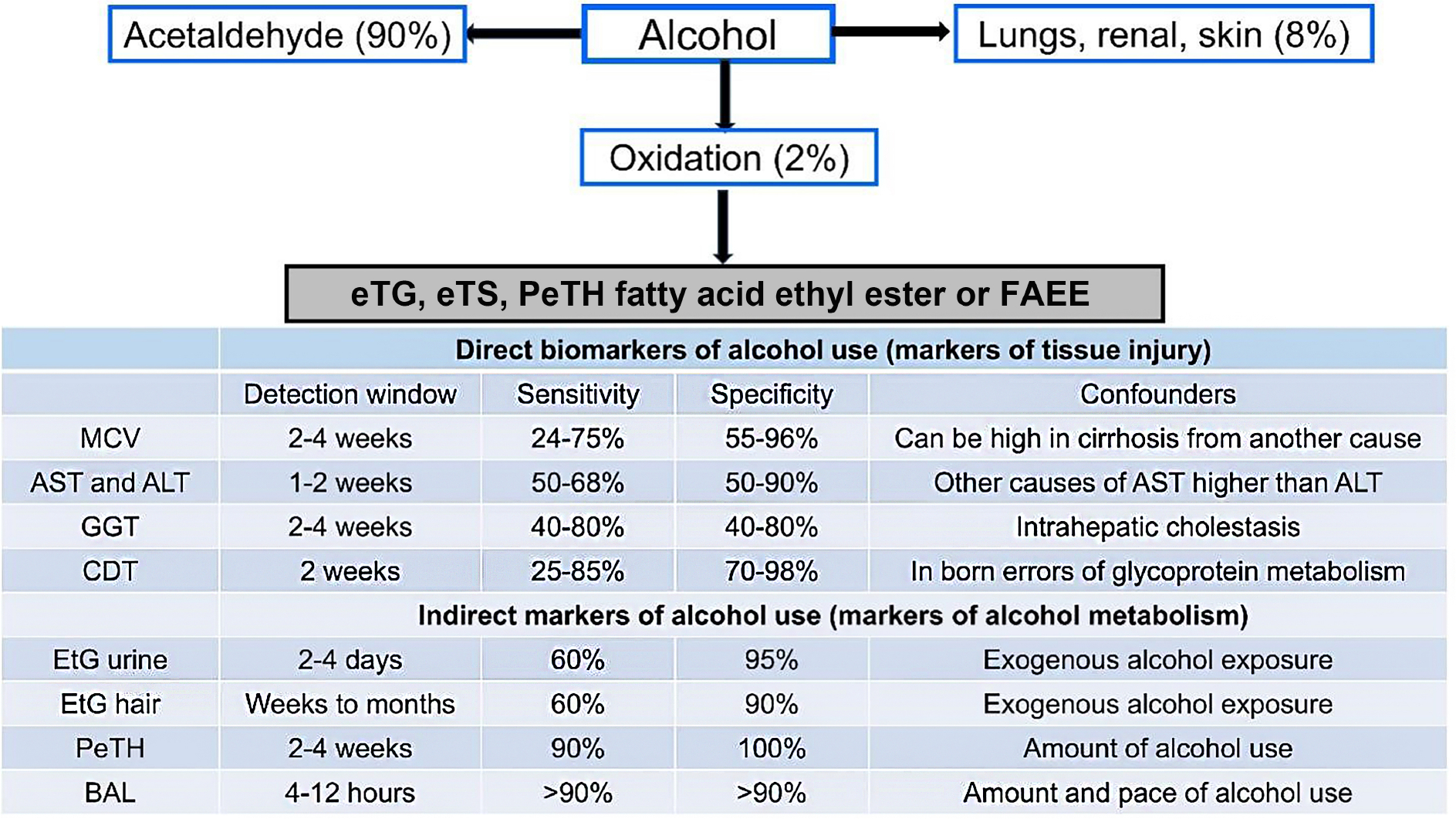
Alcohol metabolism pathways (Figure) and markers of alcohol consumption (Table). BAL: Blood alcohol level; CDT: carbohydrate-deficient transferrin; eTG: ethyl glucuronide; eTS: ethyl sulfate; FAEE: fatty acid ethyl ester; GGT: gamma-glutamyl transferase; MCV: mean corpuscular volume; PeTH: phosphatidylethanol; AST: aspartate aminotransferase; ALT: alanine aminotransferase.

Several screening tools are available to assess individuals’ alcohol consumption and its effects on their lives [Table 2]. AUDIT (Alcohol Use Disorders Identification Test) is a validated 10-item questionnaire (0–4 scale) with a sensitivity of 64%−86% and a specificity of 74%−94% for detecting alcohol use in the past 12 months. A total score of > 8 out of a maximum possible 40 indicates an AUD^[1]^. A score of 15 suggests severe AUD and a score of 20 indicates alcohol dependence, typically warranting referral to a mental health specialist [Figure 2]. Brief interventions (such as counseling patients about the harmful health effects of alcohol) are recommended for all individuals with AUD and may be sufficient for those with mild to moderate AUD (AUDIT score < 15)^[25]^. AUDIT-C, a shorter version consisting of the first three AUDIT questions, provides similar accuracy in identifying AUD, with a cut-off score of ≥ 3 for females and ≥ 4 for males (out of a maximum score of 12)^[26]^. Additionally, the DSM-V criteria classify AUD based on lifetime symptoms as mild (2–3 symptoms), moderate (4–6 symptoms), and severe AUD (7–11 symptoms)^[27]^.

The timeline follow-back (TLFB) tool is another validated method for assessing average daily alcohol consumption over a specific period, ranging from 7 days to 24 months. An online version is available for cases where in-person visits are not feasible. TLFB has been validated for assessing alcohol use in liver transplant recipients^[28]^, enhances patient motivation to change^[29]^, and quantifies alcohol consumption according to WHO risk levels: abstinence as no alcohol use; low risk: 1–40 g/day for males and 1–20 g/day for females; moderate risk:41–60 g/day for males and 21–40 g/day for females; high risk: 61–100 g/day for males and 41–60 g/day for females; very high risk: > 100 g/day for males and > 60 g/day for females.

Healthcare providers should use open-ended questions and adopt an empathetic approach, maintain strong eye contact, and use non-judgmental verbal and non-verbal communication. Avoiding any negative comments can help build rapport with patients and encourage accurate self-reporting of alcohol use.

#### Biomarkers of alcohol use

Biomarkers provide objective data to supplement self-reported alcohol use. While direct biomarkers of alcohol-related tissue injury are less expensive, their clinical utility is limited due to low accuracy. Indirect biomarkers, such as ethyl glucuronide and phosphatidylethanol, which are by-products of the non-oxidative metabolism of alcohol [Figure 2], offer greater reliability, and several are widely available for clinical use^[30]^.

**Figure 3.**
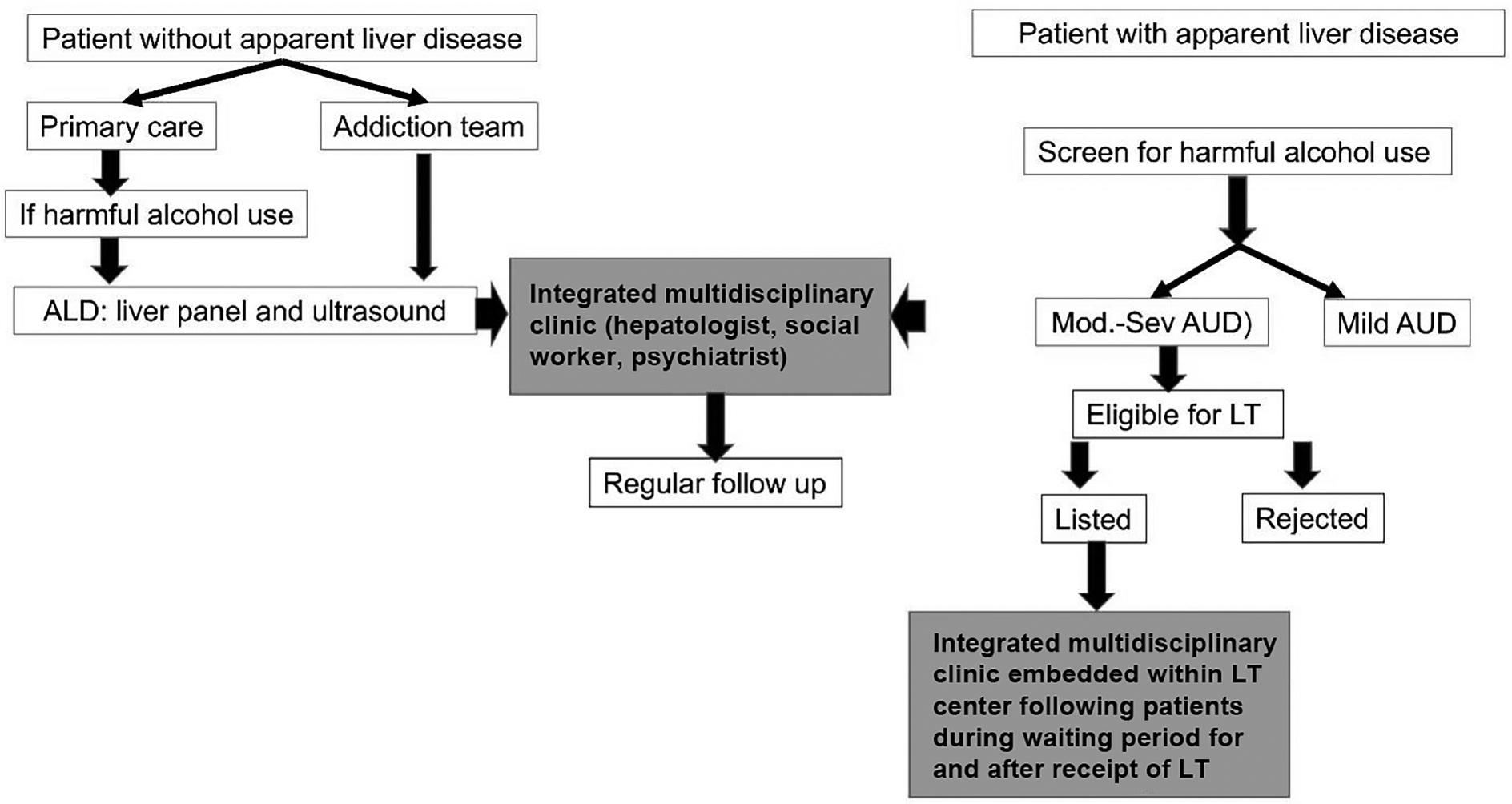
Integrated care model with a multidisciplinary approach with hepatology and addiction medicine specialists seeing patients together in the same clinic. Within the liver transplant setting, the addiction team is embedded within the transplant program and follows patients through evaluation, waiting period, and after liver transplantation. AUD: Alcohol use disorder; ALD: alcohol-related liver disease; LT: liver transplantation.

Healthcare providers should assess alcohol use at every patient visit. Individuals identified as at risk for alcohol misuse should undergo further evaluation for ALD to facilitate earlier intervention and reduce the risk of progression to advanced liver disease, including cirrhosis and its complications [Figure 3]. Academic institutions play a crucial role in ensuring future hepatologists and gastroenterologists receive formal and practical training in addiction medicine, equipping them with the skills necessary to promptly identify and manage AUD in patients with ALD^[22,24]^.

## TREATMENT OF AUD IN ALD PATIENTS

### Behavioral therapies

Behavioral therapies, administered by licensed and experienced professionals, include interventions such as motivational interviewing (MI) and cognitive behavioral therapy (CBT). MI uses patient-reported information to help individuals recognize their alcohol use problem, resolve ambivalence about control, and build motivation for behavior change^[31,32]^. CBT, on the other hand, focuses on modifying patients’ thoughts and behaviors related to alcohol consumption.

These therapies can be delivered in various settings, including outpatient programs, intensive structured outpatient treatment (at least 9 h per week), medically monitored inpatient treatment, or inpatient treatment with active medical management [Figure 3]^[33,34]^.

### Pharmacological therapies

Behavioral treatment is essential for the treatment of AUD, while MAUD can supplement the management in more severe cases. Naltrexone, acamprosate, and disulfiram are Food and Drug Administration (FDA)-approved MAUD. However, there is currently no clinical data supporting the efficacy and safety of these medications in ALD patients. A recent retrospective study involving 100 ALD patients treated with naltrexone for AUD assessed safety based on liver enzyme changes and found that naltrexone is safe for patients with compensated cirrhosis^[35]^. However, randomized controlled trials (RCTs) are needed to evaluate the safety and efficacy of FDA-approved MAUD in ALD patients.

Among the non-FDA-approved MAUD [Table 3]^[36–38]^, baclofen has been studied in ALD patients, including those with decompensated cirrhosis and/or AH^[39]^. Both baclofen and gabapentin have been endorsed to treat AUD in ALD patients by the American College of Gastroenterology and the American Psychiatry Association, respectively.

Pharmacotherapy may also be necessary for managing psychiatric comorbidities^[38]^. The prevalence of such comorbidities among AUD patients is as high as 40%−60%, especially in those with concomitant use of other recreational drugs^[40,41]^.

### Real-world scenario of AUD treatment in ALD patients

In clinical practice, AUD is treated in only 10%−30% of ALD patients, with pharmacotherapy being even much rarer, administered to just 1%−10% of cases, notably including those with cirrhosis^[42,43]^. For example, a retrospective cohort study of Medicaid beneficiaries with AUD revealed that in 2019, only 25% received treatment for AUD, and just 8.1% received pharmacotherapy. Beyond patient-related and healthcare infrastructure barriers^[44,24]^, significant clinician-related obstacles include the perception of inadequate training in addiction medicine and insufficient clinic time to simultaneously address both ALD- and AUD-related issues.

Observational studies indicate that treating AUD in patients with ALD is associated with several benefits, including: (1) improved liver-related outcomes (such as reduced liver decompensation in advanced ALD and lower risk of cirrhosis development in early ALD stages; (2) decreased hospital readmissions; and (3) enhanced survival rates and patient-reported quality of life^[7,8,42,45]^. Limited but high-quality data from four RCTs involving 346 ALD patients suggest that MAUD reduce alcohol consumption by 32%, *P* = 0.03. Furthermore, adverse effects associated with MAUD were reported in only 3% of cases^[46]^. To overcome barriers to AUD treatment and provide comprehensive, integrated care for ALD patients, federal policies are needed to support the broader implementation of AUD management strategies.

## INTEGRATED CARE MODEL FOR MANAGEMENT OF ALD PATIENTS

The monitoring and treatment of AUD in ALD patients can be approached in two ways: either by hepatologists and psychiatrists seeing patients in their respective practices simultaneously (non-integrated model), or preferably within an integrated model embedded within a liver clinic (for ALD patients outside LT setting) or within a LT center (for ALD patients listed for transplant, awaiting LT, or post-transplant recipients)^[10,47,48]^ [Figure 1].

Several observational studies have demonstrated the superiority of the integrated care model over the non-integrated approach for managing AUD in ALD patients. However, these studies have predominantly been conducted within the LT setting, mostly among LT recipients, including those receiving early LT for severe AH^[49–52]^. For example, an observational study by Addolorato *et al*. compared 55 LT recipients from 2002 onwards, treated using an integrated model, with 37 transplant recipients prior to 2002 who did not receive integrated care. This study found that the group treated with the integrated model had a significantly lower relapse rate (16.4% *vs*. 35.1%, *P* = 0.038) and better long-term survival (84.5% *vs*. 62.2%, *P* = 0.01)^[50]^. Pooled data from six studies involving 649 LT recipients showed that the integrated care approach for AUD treatment was associated with a 44% reduction in the odds of alcohol relapse and a 71% reduction in patient mortality^[43]^. The integrated care model also helps build trust between patients and their healthcare providers, benefiting both patients and healthcare systems^[51]^. It has been shown to reduce healthcare costs for payers, organizations, and healthcare systems alike^[45,53]^.

A collaborative, multidisciplinary approach to care has proven beneficial in the management of various diseases. In hepatology, the assessment for LT candidacy is an exemplary model of integrated, multidisciplinary care. Although several societies recommend its implementation for ALD patients^[54–57]^, multidisciplinary integrated care is very rarely performed in routine clinical practice. Even within LT settings, the integrated approach starts during the evaluation for LT eligibility, and proceeds during follow-up care once the hepatologist has decided to list a patient for transplantation. However, this is not the case in all centers.

By empowering hepatologists and mental health specialists to address and manage issues specific to their respective specialties in a given patient, the integrated care model helps reduce patient-perceived stigma by minimizing the need for patients to attend separate mental health centers^[10]^. Clearly, there is a pressing need to implement and promote the multidisciplinary care model for managing ALD patients. Further prospective studies should compare integrated *vs*. non-integrated AUD care in ALD patients. Additionally, RCTs evaluating the integrated treatment of both AUD and ALD are needed to assess the effects of reduced alcohol consumption on long-term patient survival and liver outcomes. Although abstinence is the primary goal for ALD patients, especially those with advanced disease, these studies may also provide valuable information on safe levels of alcohol consumption.

### Structure of the integrated multidisciplinary model

Integrated care teams consist of liver specialists, mental health professionals, counselors, and social workers. Each team member meets separately with the patient in the liver clinic to develop a comprehensive management and follow-up plan. It is important that team members bring their respective expertise and engage in constructive, patient-focused feedback. This approach maximizes the contributions of each member, ensuring optimal outcomes.

The structure of the Integrated Multidisciplinary Model can be adapted to accommodate resource and workforce limitations. For instance, if a psychiatrist is unavailable for in-person consultation, the model could be adjusted to allow for remote psychiatric input regarding MAUD or medications for concomitant psychiatric issues, via phone discussions with the patient and other team members. Similarly, the model should be flexible enough to offer telehealth services (or other virtual communication methods) when the patient is unable to attend in person. Any modifications to the model and approach should be discussed and agreed upon by the entire team.

#### Specific roles of team members

Hepatologist - provides a thorough assessment to confirm the diagnosis and determine the severity of liver disease^[58]^, identifies liver-related complications, evaluates nutritional status, and assesses the need for LT^[59]^. When following up with LT recipients, the hepatologist monitors graft function, investigates potential causes of abnormalities, manages immunosuppression, and ensures appropriate health maintenance for immunosuppressed individuals.

**Figure 4.**
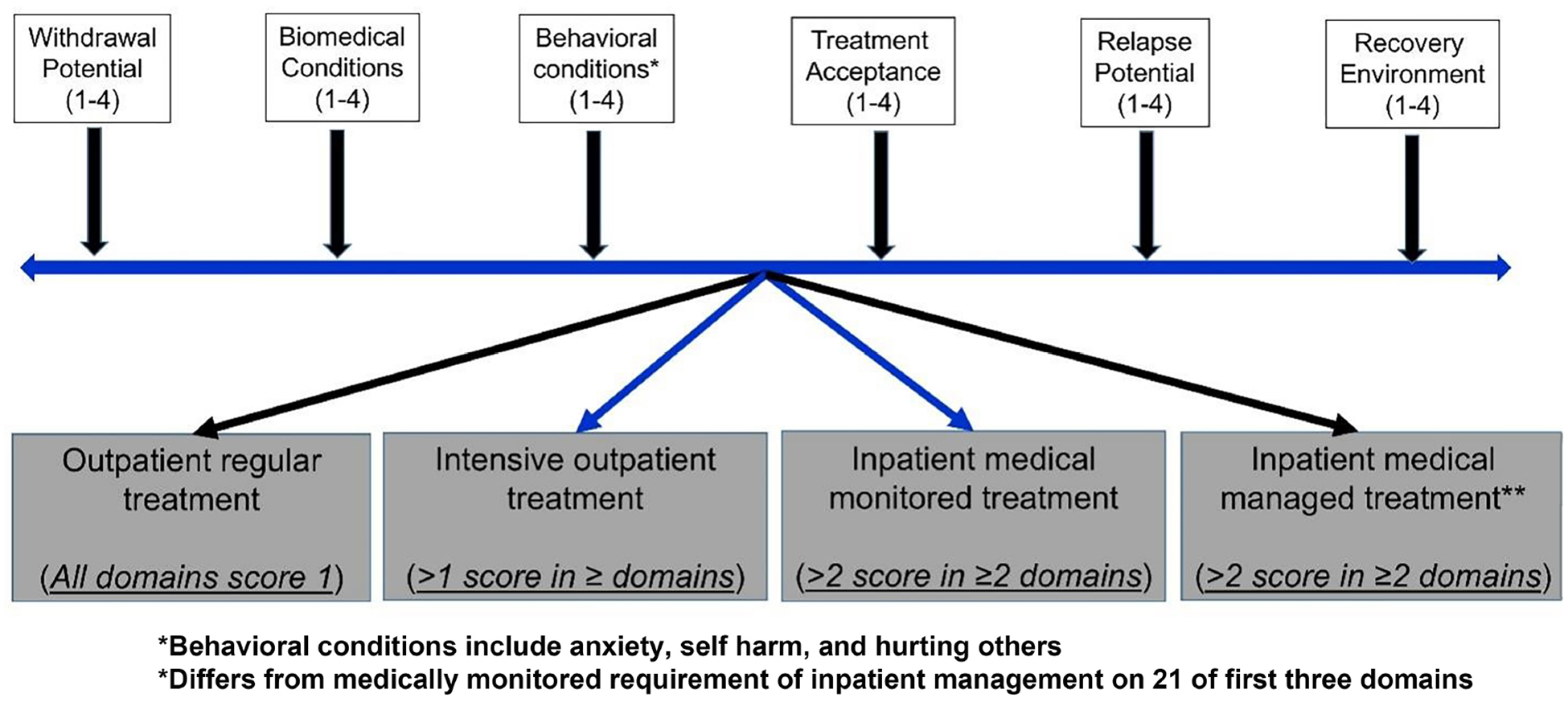
Using the six risk domains for alcohol relapse, treatment for alcohol use disorder stratification as per American Society of Addiction Medicine recommendations.

Addiction counselor - identifies alcohol use and AUD, along with any barriers to treatment and motivation to change. The goal of the assessment is to develop an ideal treatment plan based on a thorough evaluation of all the specific domains outlined by the American Society of Addiction Medicine (ASAM) [Figure 4]^[60]^. Regular follow-up and comprehensive assessment help ensure continuity of care and smooth transitions^[33,34]^. Although alcohol abstinence is the ultimate goal, a reduction of at least two levels on the WHO risk stratification scale is considered a successful treatment outcome in AUD patients. Prospective studies are needed to determine whether this definition of success also applies to patients with ALD. Remission can be defined as the absence of DSM-5 criteria for ≥ 3 months, and remission is considered sustained if these criteria are not met for ≥ 12 months.

Social worker - assesses the psychosocial aspects of patients, helping them set realistic goals for success^[61]^. The social worker provides resources to assist in achieving these defined goals and coordinates patient management between clinic visits^[62]^.

Psychiatrist - assesses psychiatric comorbidities (such as depression, anxiety, and any others), provides pharmacotherapy for AUD and psychiatric comorbidities, identifies high-risk patients for withdrawal, implements preemptive management, and addresses alcohol withdrawal symptoms when they occur^[63]^.

## SUMMARY AND FUTURE PROSPECTS

ALD is the most common liver condition and significantly contributes to the healthcare burden. Healthcare providers should screen for alcohol use during every medical encounter. Individuals identified as engaging in at-risk alcohol consumption (> 20 g/d for females and > 30 g/d for males) should be further screened for ALD and its severity. Healthcare providers taking care of ALD patients should identify the dual pathology of liver disease and AUD to ensure comprehensive management and improve long-term outcomes. An integrated multidisciplinary approach, involving hepatologists, psychiatrists, addiction counselors, and social workers, should be promoted to provide holistic care for ALD patients. Furthermore, clinical research is needed to address knowledge gaps and unmet clinical needs [Table 4] in the management of patients with ALD.

## Figures and Tables

**Table 1.** DSM-5 criteria for diagnosis of alcohol use disorder

1.	Drinking more or longer than intended?
2.	Unsuccessful desire or attempt to decrease alcohol use
3.	Spent a lot of time drinking? Or being sick or getting over other side effects?
4.	Wanted a drink so badly you couldn’t think of anything else?
5.	Found that drinking often interfered with taking care of your home, family, or job?
6.	Continued to drink even though it was causing trouble with your family or friends?
7.	Given up or cut back on activities that were important or pleasurable to you?
8.	Got into situations more than once when drinking increased your chance of getting injured or having unsafe sex?
9.	Continued drinking despite feeling depressed or anxious or memory blackout?
10.	Have you had to drink more to get the effect you want?
11.	Experienced withdrawal symptoms (insomnia, tremors, restlessness, nausea, sweating, tachycardia, or a seizure?) if not drinking for a few hours or days?

Modified from https://www.niaaa.nih.gov/publications/brochures-and-fact-sheets/alcohol-use-disorder-comparison-between-dsm. DSM-5: Diagnostic and Statistical Manual of Mental Disorders, Fifth Edition.

**Table 2.** Screening tools to screen for alcohol use

Tool	Components	Comments
SASQ	How many times have you had > 5 (for males) or > 4 (for females) drinks in the past year?	If yes, then use AUDIT or AUDIT-C Sensitivity 82%−87%, specificity 61%−80%
AUDIT-C (alcohol use disorder identification test-consumption)	First 3 questions of full AUDIT tool	Of a maximum score of 12, > 3 in males > 2 in females Limitation is low, specificity of about 60%
AUDIT	10-item questionnaire on alcohol use over the previous 12 months	Of a maximum score of 40, ≥ 8 identifies AUD with cut-offs 15 and 20 stratified to severe AUD and dependence
DSM 5	11 criteria [Table 1] on physical, social, professional, and interpersonal effects of alcohol use	Needs at least 2 criteria for AUD diagnosis
CAGE	(1) Have you ever felt you should Cut down on your drinking? (2) Have people Annoyed you by criticizing your drinking? (3) Have you ever felt Guilty about your drinking? (4) Eye-opener: have you ever had a drink first thing in the morning to steady your nerves?	2 indicates AUD, but only 56% accurate in identifying active alcohol abuse
MAST	List of 25 questions (total score from 0 to 53)	A score > 4 identifies AUD. SMAST with 13 questions is as sensitive for diagnosis Cannot differentiate between current and past drinking
FAST, VAST, and PAT	Short questionnaire	Useful in quick lifetime risk of alcohol dependence

SASQ: Single alcohol screening question; AUDIT: Alcohol Use Disorders Identification Test; AUD: alcohol use disorder; DSM-5: Diagnostic and Statistical Manual of Mental Disorders, Fifth Edition; MAST: Michigan alcohol screening test; SMAST: shorter version Michigan alcohol screening test; FAST: fast alcohol screening test; VAST: veterans alcoholism screening test; PAT: Paddington alcohol test; CAGE: Cut Annoyed Guilty Eye opener.

**Table 3.** Pharmacological therapies for AUD

	Mechanism of action	Dose recommended	Target outcome	Notes
**FDA-approved medications**				
Naltrexone	Mu opioid receptor antagonist	50 mg once a day orally 380 mg intramuscular once a month	Achieve and maintain abstinence and reduce drinking	Start 25 mg/d and increase to 50 mg/d Cautious use in decompensated cirrhosis
Acamprosate	NMDA receptor agonist to modulate glutamate activity	666 mg tid orally	Achieve and maintain abstinence	Dosing and pill burden limit compliance No hepatic metabolism
Disulfiram	Inhibits aldehyde dehydrogenase	250–500 mg once a day orally	Achieve and maintain abstinence	Caution with the history of psychosis
**Non-FDA approved medications**				
Varenicline	Nicotinic acetylcholine receptor agonist	Up to 1 mg twice a day orally	Reduce drinking (particularly useful for smokers)	Hepatic metabolism and avoid in those with any spectrum of liver disease
Topiramate	Enhances GABA activity via inhibiting glutamate	Up to 300 mg/d orally in divided doses	Achieve abstinence, reduce drinking, and craving outcomes	Dose-dependent cognitive issues mimic hepatic encephalopathy Partial hepatic metabolism
Gabapentin	Modulates GABAergic transmission	900 mg twice a day orally (maximum dose up to 3.6 gm per day in 3 divided doses)	Achieve abstinence and reduce drinking	Caution with opioid use disorder No hepatic metabolism
Baclofen	GABA_B_ agonist	10–20 mg 3 times daily orally (maximum dose 80 mg daily)	Achieve and maintain abstinence	Minimal hepatic metabolism
Ondansetron	5-HT3 receptor antagonist	1–16 mcg/kg twice daily	Achieve abstinence and reduce drinking	Useful in combination with naltrexone Reports of liver toxicity are documented

AUD: Alcohol use disorder; NMDA: N-methyl-D-aspartate; FDA: Food and Drug Administration; GABA: gamma-aminobutyric acid.

**Table 4.** Areas of clinical unmet need and of future research opportunities in patients with ALD

1.	Randomized integrated design clinical trials using liver and AUD-directed targets
2.	Examine the beneficial effects of AUD treatment by comparing integrated and non-integrated models of care
3.	Examine the cost-effectiveness of the treatment of AUD in ALD patients
4.	Derive strategies to overcome barriers that limit the treatment of AUD
5.	Derive a safe level of alcohol use
6.	Define endpoints on alcohol use as a surrogate for patient outcomes
7.	Derive data on biomarkers of alcohol use like Peth for stratification of subtypes of steatotic liver disease
8.	Develop objective criteria and a protocol for selecting patients for early liver transplantation
9.	Define the recurrence of alcohol use among liver transplant recipients
10.	Evaluate the role of PeTH in candidates evaluated for or awaiting liver transplant in reducing post-transplant alcohol use

AUD: Alcohol use disorder; ALD: alcohol-related liver disease.
